# Lipid Changes During Endocrine Therapy in Breast Cancer Patients: The Results of a 5-Year Real-World Retrospective Analysis

**DOI:** 10.3389/fonc.2021.670897

**Published:** 2022-01-17

**Authors:** Tao He, Xu Li, Jiayuan Li, Zhu Wang, Yuan Fan, Xiusong Li, Zhoukai Fu, Yunhao Wu, Qing Lv, Ting Luo, Xiaorong Zhong, Jie Chen

**Affiliations:** ^1^ Department of Breast Surgery, West China School of Medicine/West China Hospital, Sichuan University, Chengdu, China; ^2^ Center of Biostatistics, Design, Measurement and Evaluation (CBDME), Department of Clinical Research Management, West China Hospital, Sichuan University, Chengdu, China; ^3^ West China School of Public Health and West China Fourth Hospital, Sichuan University, Chengdu, China; ^4^ Laboratory of Molecular Diagnosis of Cancer, West China Hospital, Sichuan University, Chengdu, China; ^5^ Department of Clinical Medicine, Fujian Medical University, Fuzhou, China; ^6^ Department of Breast Surgery, Clinical Research Center for Breast Diseases, West China Hospital, Sichuan University, Chengdu, China; ^7^ West China School of Medicine/West China Hospital, Sichuan University, Chengdu, China; ^8^ Department of Head and Neck and Mammary Gland Oncology, Cancer Center, West China Hospital, Sichuan University, Chengdu, China; ^9^ Department of Medical Oncology, Cancer Center, West China Hospital, Sichuan University, Chengdu, China

**Keywords:** breast cancer, endocrine therapy, lipids, tamoxifen, letrozol, anastrozole, exemesatne

## Abstract

**Background:**

The aim of this study was to investigate the status of serum lipids during endocrine therapy.

**Methods:**

We retrospectively analysed lipid profiles during the 5-year treatment of 1487 consecutive postoperative BC patients. Lipid parameters included triglycerides (TG), total cholesterol (TC), low-density lipoprotein (LDL-C) and high-density lipoprotein (HDL-C). Those biomarkers were measured at baseline and 1, 2, 3, 4 and 5 years following the initiation of endocrine therapy.

**Results:**

For premenopausal BC patients, LDL levels rapidly decreased at 1 year in the tamoxifen (TAM) group compared with baseline levels (*p*<0.05), and this decline remained for the following 4 years. Additionally, LDL levels were significantly lower in the TAM group than in the nonendocrine group at all assessment time points (*p*<0.05). Similarly, TC levels also decreased in the TAM group compared with baseline levels at all assessment time points (*p*<0.05), and compared with the levels in the nonendocrine group, TC levels were also lower for the first 4 years. For postmenopausal BC patients, there was no significant difference in the lipid profiles (TG, TC, LDL and HDL) in the letrozole (LET), anastrozole (ANA) or exemestane (EXE) groups compared with the nonendocrine group. For patients who received TAM, compared with the nonendocrine group, TC levels decreased at 1 year, and LDL levels decreased at 1 and 2 years.

**Conclusions:**

TAM may improve LDL and TC levels in premenopausal BC patients. In postmenopausal BC patients, aromatase inhibitors (AIs) may have no adverse effects on lipid profiles, and TAM may have limited beneficial effects on serum lipids.

## Background

Breast cancer (BC) is one of the most common malignancies, with an incidence of approximately 2100000 new cases and a mortality of approximately 630000 worldwide in 2018 (1, 2). Different BC subtypes have different biological behaviours and clinical prognoses. Among these, hormone receptor (HR)-positive BC, accounting for 65%-75% of BC cases, is the most common type deserving urgent exploration (3–5). Extensive studies have shown that the better prognosis of HR-positive BC patients may be due to adjuvant endocrine therapy, which substantially reduces recurrence rates and improves overall survival (6).

Prior studies have demonstrated that oestrogen plays a vital role in the development and progression of HR-positive BC (7, 8), and thus, deregulation of oestrogen signalling may be the basic therapeutic strategy for endocrine therapy. Endocrine drugs include selective oestrogen receptor modulators (SERMs) and aromatase inhibitors (AIs) (9, 10). As a first-generation SERM, tamoxifen, which was applied in the 1970s, blocks oestrogenic effects by antagonizing oestrogen binding to oestrogen receptors (ERs) and interfering with receptor-mediated transcriptional events (11). AIs (including letrozole, anastrozole and exemestane) inhibit the aromatase enzyme, which is the rate-limiting enzyme in the conversion of androgens to oestrogens, thus leading to the reduction of plasma and intratumoural oestrogen levels (12, 13).

For BC patients receiving endocrine therapy, the oestrogen level might be reduced due to the ovarian function decrease and the side effects of endocrine drugs. While, oestrogen possesses several physiological functions, including involvement in bone and lipid metabolism as well as cardiovascular, cognitive and sexual functions (14). Thus, the decrease in oestrogen may cause some side effects during the endocrine therapy period. In particular, endocrine therapy lasts for 5-10 years (15), and the long-term potential adverse effects of endocrine therapy deserve exploration. Beneficial effects of oestrogen on lipid metabolism have been widely reported. Additionally, previous studies have revealed that the decrease in oestrogen is associated with an increased rate of hyperlipidaemia and cardiovascular disease (CVD), including myocardial infarction and stroke (16, 17). Based on the latest available statistics, with the increasing survival rate of BC patients, the proportion of non-cancer-related death has increased gradually. CVD, accounting for 35% of non-cancer-related deaths in BC patients, is the most common cause of non-cancer-related death and even competes with BC as the leading cause of death in older patients with early BC (18). Thus, possible long-term consequences of endocrine treatment-related changes in lipid metabolism are gradually drawing increasing attention in BC patients receiving endocrine therapy.

To date, there has been little agreement about the role of endocrine drugs on lipid metabolism (19–21). Thus, in the present study, we retrospectively investigated the changing trend of lipid profiles during the entirety of endocrine treatment and explored the effects of different endocrine drugs on lipid metabolism in HR-positive BC patients.

## Materials and Methods

### Patient Selection

From February 2009 to January 2015, the medical records of all consecutive BC patients were retrospectively collected from the Department of Breast Surgery, West China Hospital, Sichuan University. The Institutional Review Board and Ethics Committee of West China Hospital approved this retrospective study (IRB No. 2019-1023). The inclusion criteria were as follows: (1) female patients aged ≥18 years; (2) patients pathologically diagnosed with BC; (3) postoperative BC patients and the method of operation were according to the NCCN guidelines (4) stage I-III BC patients; (5) postoperative systemic therapies including endocrine therapy, chemotherapy, targeted therapy, and radiotherapy were recommended according to the NCCN guidelines, (6)patients who did not receive endocrine therapy or patients who received one of the following endocrine drugs: tamoxifen (TAM), letrozole (LET), anastrozole (ANA), or exemestane (EXE); and (7) patients who had adequate organ function before surgery and Eastern Cooperative Oncology Group (ECOG) ≤2. The exclusion criteria were as follows: (1) patients with bilateral BC; (2) patients with inflammatory BC; (3) patients with tumors in other systems; (4) patients who received endocrine therapy before being diagnosed with BC; (5) patients who had not completed the established endocrine regimens or who had changed endocrine drugs for various reasons; (6) patients diagnosed with dyslipidemia and taking lipid-lowering drugs before endocrine therapy; (7) patients with heart disease and liver dysfunction before endocrine therapy; and (8) pregnant and lactating women.

### Data Collection and Evaluated Parameters

All clinicopathological data were reviewed from medical records by two clinicians who signed confidentiality agreements. The variables of interest included height, weight, body mass index (BMI), age at diagnosis, menopausal status, tumour location, surgical procedures, TNM (Tumour, Node, Metastasis) stage (22), ER, progesterone receptor (PR), human epidermal growth factor receptor-2 (HER-2), Ki-67 status and lipid parameters at baseline (within one week prior to drug administration) and at 1, 2, 3, 4 and 5 years following the initiation of endocrine therapy. Height and weight were assessed for the determination of BMI, which was calculated as the weight divided by the height squared (kg/m^2^). Lipid parameters includes triglycerides (TG), total cholesterol (TC), high-density lipoprotein (HDL) and low-density lipoprotein (LDL). All patients fasted overnight before blood sampling, and blood samples were collected the next morning on an empty stomach. To avoid lipid changes associated with freezing and storage, all lipid analyses were carried out from unfrozen samples within 2 hours after sampling. All measurements were performed on a Cobas8000 system (Roche Diagnostics GmbH, Germany) in the Department of Laboratory Medicine, West China Hospital, Sichuan University, which is a College of American Pathologists (CAP)-accredited laboratory. According to the Chinese guidelines for the management of dyslipidaemia (23), dyslipidaemia was considered if patients met at least one of the following criteria: TG≥ 1.7 mmol/L, TC ≥ 5.2 mmol/L, LDL-C ≥ 3.4 mmol/L or HDL-C ≤ 1.0 mmol/L.

### Endocrine Drugs

For premenopausal patients, the nonendocrine group (ER and PR negative) did not receive any endocrine therapeutic agents, while the TAM treatment group (ER or PR positive) received 5-year continuous oral administration of 10 mg TAM twice daily.

Similarly, for postmenopausal patients, the nonendocrine group (ER and PR negative) did not receive any endocrine therapeutic agents, while ER or PR positive patients received continuous oral administration of one of the following endocrine agents: TAM (20mg/d), LET (2.5 mg/d), ANA (1 mg/d) or EXE (25 mg/d). Medication was begun within 2 weeks of the end of chemotherapy treatment and planned to continue for 5 years.

### Statistical Analysis

According to the menopausal status, the participants were stratified into premenopausal and postmenopausal groups. The differences in baseline lipid profiles and the baseline covariates in several endocrine therapy groups were evaluated by ANOVA and the chi-square test.

The generalized linear mixed model was used to evaluate the changing rules of lipid profiles in different endocrine therapy groups during the treatment period. First, the time of administration was taken as a continuous variable to evaluate the overall trend of blood lipid changes with time and the influence of different endocrine agents on blood lipids in both premenopausal and postmenopausal patients. In both premenopausal and postmenopausal subgroups, BMI, smoking, hypertension, age, and chemotherapy, endocrine therapy medication type, medication time (classified or continuous), and medication type * time were taken as the fixed effects, the intercept was taken as the random effect, and the UN covariance matrix (unstructured covariance) was used. Since the distribution of postmenopausal baseline LDL in the different endocrine groups was significantly different, in the analysis of postmenopausal LDL, the difference between LDL levels at the assessment time points and baseline LDL levels were used as the response variables of the generalized linear mixed model. Then, the time of administration was taken as a classified variable to explore the changes in lipid profiles in different medication groups and the differences among different medication groups at 1, 2, 3, 4, and 5 years. The trend of serum lipid levels in premenopausal and postmenopausal groups and the blood lipid levels of different AI groups were described by model adjusted least-square means.

All the analyses in this study were carried out with SAS 9.4 software (SAS Institute, Cary, NC, USA). Statistical tests were all 2-sided and *p* < 0.05 was considered statistically significant.

## Results

### Patient Characteristics

**Figure 1:**
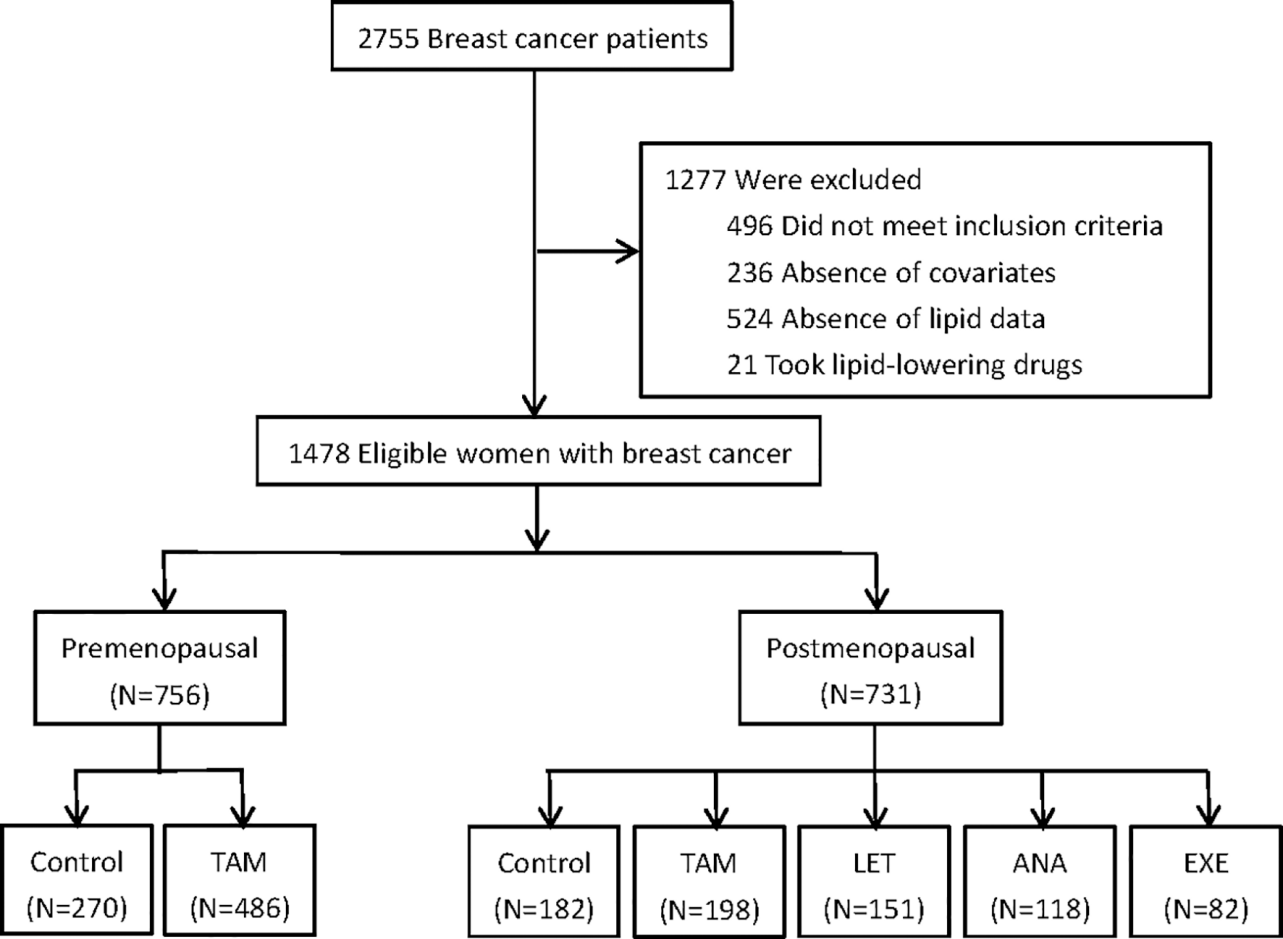
Flow chart of included and excluded patients.

A total of 1487 eligible and consecutive postoperative BC patients were identified for this study, with 756 premenopausal patients and 731 postmenopausal patients (**Figure 1**). The clinicopathological characteristics are summarized in **Tables 1**, **2**. For the premenopausal women, 486 patients received TAM treatment, and 270 patients did not receive endocrine drugs. For the postmenopausal patients, 198 patients received TAM, 151 patients received LET, 118 patients received ANA, 82 patients received EXE, and 182 patients did not receive endocrine therapy.

**Table 1:** Baseline characteristics of premenopausal breast cancer patients.

VARIABLE	Control (n = 270)	TAM (n = 486)	F/X^2^	P
Age, mean (SD)	42.83 (5.90)	42.36 (5.22)	1.35	0.25
Smoking, n (%)			1.00	0.30
Yes	1 (0.37%)	5 (1.03%)		
No	269 (99.63%)	481 (98.97%)		
Hypertension, n (%)			0.04	0.83
Yes	5 (1.85%)	8 (1.65%)		
No	265 (98.15%)	478 (98.35%)		
BMI, mean (SD)	22.78 (2.83)	22.95 (3.34)	0.50	0.48
Surgery, n (%)			5.72	0.06
Mastectomy	25 (9.26%)	59 (12.14%)		
Breast conserving operation	235 (87.04%)	392 (80.66%)		
Breast reconstruction	10 (3.70%)	35 (7.20%)		
Chemotherapy, n (%)			5.02	0.08
anthracycline-plus-taxane based regimens	124 (45.93%)	208 (42.80%)		
anthracycline-based regimens	34 (12.59%)	92 (18.93%)		
taxane-based regimens	112 (41.48%)	186 (38.27%)		
TNM stage, n (%)			0.68	0.71
Stage I	87 (32.22%)	144 (29.63%)		
Stage II	176 (65.19%)	331 (68.11%)		
Stage III	7 (2.59%)	11 (2.26%)		
Baseline TC level, mean (SD)	4.97 (0.86)	4.89 (0.96)	1.41	0.24
Baseline TG level, mean (SD)	1.73 (0.98)	1.71 (1.12)	0.09	0.77
Baseline LDL level, mean (SD)	3.00 (0.70)	2.93 (0.74)	1.62	0.20
Baseline HDL level, mean (SD)	1.43 (0.38)	1.45 (0.36)	0.43	0.51

**Table 2:** Baseline characteristics of postmenopausal breast cancer patients.

VARIABLE	Control (n = 182)	TAM (n = 198)	LET (n = 151)	ANA (n = 118)	EXE (n = 82)	F/X^2^	P
Age, mean (SD)	57.12 (6.53)	53.35 (6.44)	58.99 (6.48)	59.18 (6.81)	61.07 (8.51)	95.09	<0.01
Smoking, n (%)						8.47	0.076
Yes	0 (0.00%)	5 (2.53%)	1 (0.66%)	1 (0.85%)	1 (1.22%)		
No	182 (100.00%)	193 (97.47%)	150 (99.34%)	117 (99.15%)	81 (98.78%)		
hypertension, n (%)						9.98	0.041
Yes	7 (3.85%)	9 (4.55%)	13 (8.61%)	14 (11.86%)	7 (8.54%)		
No	175 (96.15%)	189 (95.45%)	138 (91.39%)	104 (88.14%)	75 (91.46%)		
BMI, mean (SD)	23.15 (3.13)	23.66 (3.00)	23.85 (3.15)	24.68 (3.58)	23.52 (2.90)	4.39	<0.01
Surgery, n (%)						15.10	0.06
Mastectomy	15 (8.24%)	3 (1.52%)	8 (5.30%)	11 (9.32%)	8 (9.76%)		
Breast conserving operation	164 (90.11%)	193 (97.47%)	142 (94.04%)	107 (90.68%)	73 (89.02%)		
Breast reconstruction	3 (1.65%)	2 (1.01%)	1 (0.66%)	0 (0.00%)	1 (1.22%)		
chemotherapy, n (%)						113.81	<0.01
anthracycline-plus-taxane based regimens	113 (62.09%)	144 (72.73%)	90 (59.60%)	71 (60.17%)	42 (51.22%)		
Anthracycline based regimens	2 (0.01%)	9 (0.05%)	1 (0.01%)	0 (0.00%)	20 (24.39%)		
Taxane based regimens	67 (36.81%)	45 (22.73%)	60 (39.74%)	47 (39.83%)	20 (24.39%)		
TNM stage, n (%)						14.03	0.08
Stage I	54 (29.67%)	54 (27.27%)	39 (25.83%)	37 (31.36%)	31 (37.80%)		
Stage II	127 (69.78%)	136 (68.69%)	111 (73.51%)	80 (67.80%)	50 (60.98%)		
Stage III	1 (0.55%)	8 (4.04%)	1 (0.66%)	1 (0.85%)	1 (1.22%)		
Baseline TC level, mean (SD)	5.22 (0.97)	5.07 (0.95)	5.24 (1.03)	5.28 (1.07)	5.19 (0.94)	1.14	0.34
Baseline TG level, mean (SD)	1.82 (1.14)	1.77 (1.05)	1.81 (0.79)	1.87 (0.87)	1.66 (0.83)	0.62	0.65
Baseline LDL level, mean (SD)	3.16 (0.85)	3.00 (0.76)	3.27 (0.79)	3.35 (0.79)	3.12 (0.83)	4.42	<0.01
Baseline HDL level, mean (SD)	1.45 (0.40)	1.39 (0.42)	1.39 (0.32)	1.42 (0.36)	1.51 (0.46)	1.96	0.10

### The Variations in Lipid Profiles in Premenopausal Patients

**Figure 2:**
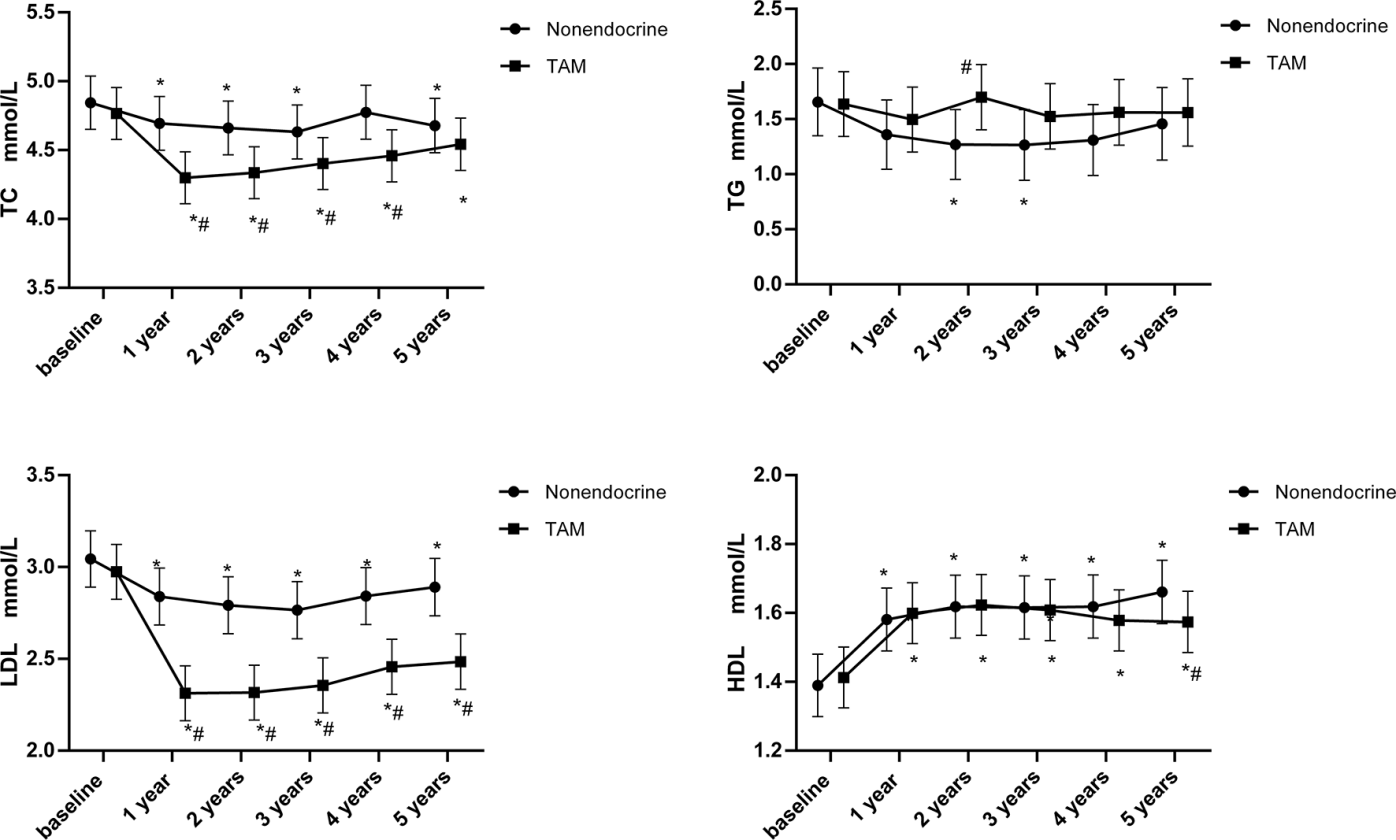
Line chart of lipid profiles during 5-years endocrine therapy in premenopausal BC patients. *p < 0.05 for comparison with baseline. #*p* < 0.05 for comparison with the nonendocrine group.

The time-dependent changes in lipid profiles and the results of intergroup tests using least-square means are shown in **Figure 2**. The means and standard error are presented for lipid parameters measured at baseline and 1, 2, 3, 4 and 5 years.

TC rapidly decreased at 1 year (*p*<0.05) and then showed a slow increasing trend that was still below the baseline values (*p*<0.05) in the TAM group. Compared with the nonendocrine group, TC was significantly lower in the TAM group in the first 1-4 years (*p*<0.05). At 5 years, TC values were still lower in the TAM group than in the nonendocrine group, but there was no statistically significant difference.

TG was stable in the TAM group. Compared with the nonendocrine groups, it was found that TG levels were higher in the TAM group than in the nonendocrine group at 2 years (*p*<0.05); nevertheless, no difference was found at other measurement time points.

LDL rapidly decreased in the TAM group compared with baseline levels at 1 year (*p*<0.05), and the decline remained for the following 4 years (p<0.05). Notably, LDL levels were significantly lower in the TAM group than in the nonendocrine group at all assessment time points (*p*<0.05).

HDL trends witch increased at 1 year and remained at high levels at the following assessment time points were similar in both groups. Only at 5 years, HDL levels were lower in the TAM group than in the nonendocrine group (*p*<0.05).

Over all, TAM had beneficial effects on LDL and TC levels during the 5-year follow-up period (**Table 3**). Compared with the nonendocrine group, patients who received TAM had lower LDL and TC levels, and with the increase in medication time, the effects of TAM on reducing LDL increased (β=-0.05, 95% CI -0.07, -0.03). In addition, we found that TC and LDL levels were positively correlated with age (β=0.03, 95% CI 0.02, 0.04 and β=0.02, 95% CI 0.01, 0.03, respectively). Similarly, the levels of TC, TG, and LDL were also positively correlated with BMI (β=0.02, 95% CI 0.001, 0.04; β=0.05, 95% CI 0.02, 0.07; β=0.03, 95% CI 0.02, 0.04, respectively), while the HDL levels were negatively correlated with BMI (β=-0.03, 95% CI -0.04, -0.02).

**Table 3:** Comparison of lipid profiles in premenopausal BC patients.

	TC β (95%CI)	TG β (95%CI)	LDL β (95%CI)	HDL β (95%CI)
ET				
control	0.00	0.00	0.00	0.00
TAM	-0.24 (-0.37,-0.12)	0.09 (-0.15,0.32)	-0.29 (-0.39,-0.19)	0.05 (-0.01,0.11)
Time	-0.02 (-0.04,-0.002)	-0.05 (-0.11,0.01)	-0.03 (-0.05,-0.02)	0.05 (0.04,0.06)
ET*Time				
control*time	0.00	0.00	0.00	0.00
TAM*time	-0.01 (-0.04,0.02)	0.04 (-0.04,0.12)	-0.05 (-0.07,-0.03)	-0.02 (-0.03,-0.01)
Chemotherapy				
anthracycline-plus-taxane-b ased regimens	0.00	0.00	0.00	0.00
anthracycline-based regimens	0.05 (-0.10,0.21)	-0.10 (-0.34,0.14)	0.04 (-0.09,0.16)	0.02 (-0.05,0.09)
taxane-based regimens	-0.05 (-0.17,0.07)	0.01 (-0.18,0.19)	-0.04 (-0.14,0.05)	0.00 (-0.06,0.07)
Smoking				
No	0	0	0	0
Yes	0.03 (-0.57,0.63)	0.22 (-0.68,1.12)	-0.11 (-0.59,0.37)	0.04 (-0.24,0.32)
Hypertension				
No	0	0	0	0
Yes	0.26 (-0.16,0.68)	-0.20 (-0.79,0.56)	0.03 (-0.30,0.37)	0.05 (-0.14,0.25)
Age	0.03 (0.02,0.04)	0.01 (-0.01,0.02)	0.02 (0.01,0.03)	0.00 (-0.003,0.01)
BMI	0.02 (0.001,0.04)	0.05 (0.02,0.07)	0.03 (0.02,0.04)	-0.03 (-0.04,-0.02)

**Figure 3:**
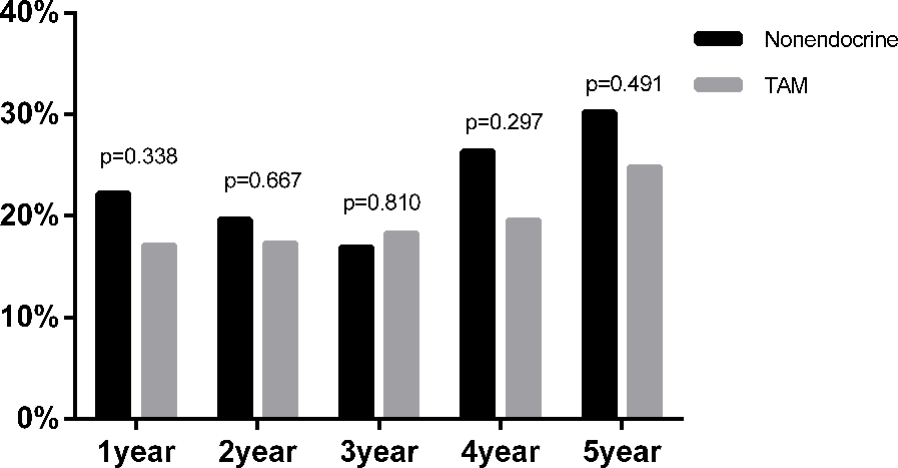
The proportion of newly diagnosed dyslipidaemia after endocrine therapy in premenopausal BC.

In addition, among the 756 premenopausal patients, 445 patients were diagnosed with dyslipidaemia (58.86%) before endocrine therapy. Moreover, the proportion of newly diagnosed dyslipidaemia during endocrine therapy in premenopausal BC was also assessed (**Figure 3**). At all assessment time points during the whole endocrine therapy period, there was no statistically significant difference in the proportion of newly diagnosed dyslipidaemia between the TAM and nonendocrine groups.

### The Variations in Lipid Profiles in Postmenopausal Patients

**Figure 4:**
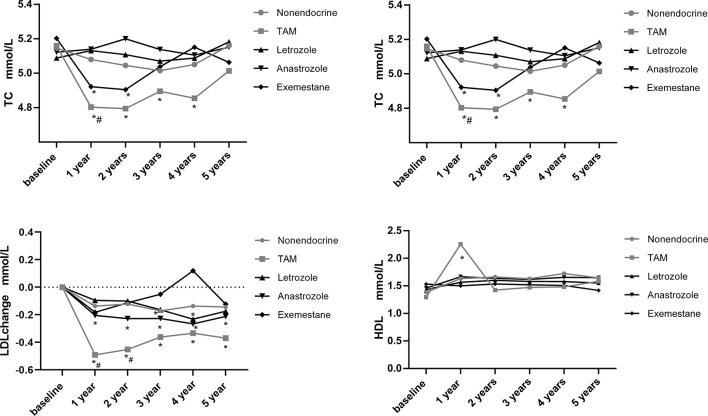
Line chart of lipid profiles during 5-years endocrine therapy in postmenopausal BC patients. *p < 0.05 for comparison with baseline. ^#^p < 0.05 for comparison with the nonendocrine group.

Additionally, we assessed the time-dependent variations in lipid profiles in postmenopausal patients, and the results are shown in **Figure 4**. The means and standard deviations for lipid parameters were still measured at baseline and at 1, 2, 3, 4 and 5 years.

TC decreased in the TAM group at 1, 2, 3 and 4 years compared with baseline levels (*p*<0.05). Additionally, TC also decreased in the EXE group at 1 and 2 years compared with baseline levels (*p*<0.05). While, TC levels remained basically stable in the LET, ANA and nonendocrine groups. We found that only at 1 year, TC levels in the TAM group were lower than those in the nonendocrine group (*p*<0.05).

TG decreased in the nonendocrine group at 2, 3, 4 and 5 years compared with baseline levels (*p*<0.05). Similarly, TG decreased in the ANA group at 1 and 4 years compared with baseline levels (*p*<0.05). Also, TG decreased in the EXE group at 1, 2 and 4 years compared with baseline levels (*p*<0.05). In the TAM and LET groups, TG levels did not change significantly. The difference between the four endocrine drugs and the nonendocrine group was not significant.

LDL decreased in both TAM and ANA groups at all assessment time points compared with baseline levels (*p*<0.05). In the LET group, LDL decreased at 4 years compared with baseline levels (*p*<0.05). Additionally, LDL levels were significantly lower at 1 and 2 years in the TAM group than in the nonendocrine group (*p*<0.05).

HDL levels increased in the TAM group at 1 year compared with baseline levels (*p*<0.05), while HDL levels did not change significantly in the LET, ANA, EXE or nonendocrine groups. Additionally, the difference between the four endocrine drugs and the nonendocrine group was not significant.

Additionally, we performed further analysis to assess the effects of endocrine drugs on serum lipids in postmenopausal patients (**Table 4**). The present study showed that there was no statistically significant alteration in the lipid profiles (TG, TC, LDL and HDL) in the LET, ANA or EXE groups compared with the nonendocrine group. For patients who received TAM, we found that LDL levels decreased from the first year, and with the increase in medication time, the effects of TAM on reducing LDL were weakened (β=0.06, 95% CI 0.02, 0.11). Additionally, BMI was positively correlated with TG (β=0.05, 95% CI 0.02, 0.07), and age was positively correlated with TC (β=0.01, 95% CI 0.00, 0.02).

**Table 4:** Comparison of lipid profiles in postmenopausal BC patients.

	TC β (95%CI)	TG β (95%CI)	LDL change β (95%CI)	HDL β (95%CI)
ET				
control	0.00	0.00	0.00	0.00
TAM	-0.14 (-0.43,0.14)	-0.01 (-0.31,0.30)	-0.50 (-0.77,-0.24)	0.14 (-0.39,0.67)
LET	-0.05 (-0.28,0.19)	-0.14 (-0.39,0.12)	-0.02 (-0.25,0.22)	0.01 (-0.48,0.51)
ANA	0.02 (-0.23,0.28)	-0.12 (-0.40,0.15)	0.03 (-0.22,0.28)	0.06 (-0.47,0.59)
EXE	-0.05 (-0.35,0.25)	-0.26 (-0.57,0.06)	-0.19 (-0.49,0.12)	0.06 (-0.54,0.66)
Time	-0.01 (-0.04,0.03)	-0.06 (-0.10,-0.03)	-0.02 (-0.05,0.01)	0.05 (-0.07,0.17)
ET*Time				
control*time	0.00	0.00	0.00	0.00
TAM*time	-0.03 (-0.08,0.01)	0.05 (-0.001,0.10)	0.06 (0.02,0.11)	-0.07 (-0.24,0.09)
LET*time	0.01 (-0.04,0.06)	0.06 (0.00,0.11)	-0.01 (-0.06,0.04)	-0.03 (-0.21,0.15)
ANA*time	0.00 (-0.05,0.06)	0.01 (-0.05,0.06)	-0.01 (-0.06,0.04)	-0.02 (-0.21,0.16)
EXE*time	0.00 (-0.06,0.06)	0.04 (-0.03,0.10)	0.05 (-0.005,0.11)	-0.07 (-0.27,0.14)
Chemotherapy				
anthracycline-plus-taxane-based regimens	0.00	0.00	0.00	0.00
anthracycline-based regimens	-0.20 (-0.53,0.13)	-0.10 (-0.45,0.25)	0.31 (0.02,0.61)	-0.05 (-0.57,0.46)
taxane-based regimens	0.16 (-0.27,0.59)	-0.07 (-0.52,0.38)	-0.32 (-0.70,0.05)	-0.13 (-0.77,0.50)
Smoking				
No	0.00	0.00	0.00	0.00
Yes	0.28 (0.00,0.55)	0.10 (-0.19,0.39)	-0.12 (-0.36,0.13)	-0.01 (-0.42,0.41)
Hypertension				
No	0.00	0.00	0.00	0.00
Yes	-0.03 (-0.25,0.19)	-0.05 (-0.29,0.18)	0.12 (-0.07,0.32)	-0.08 (-0.42,0.25)
Age	0.01 (0.00,0.02)	0.00 (-0.01,0.01)	-0.001 (-0.01,0.01)	0.00 (-0.02,0.01)
BMI	-0.00 (-0.03,0.02)	0.05 (0.02,0.07)	0.01 (-0.01,0.03)	-0.03 (-0.06,0.01)

**Figure 5:**
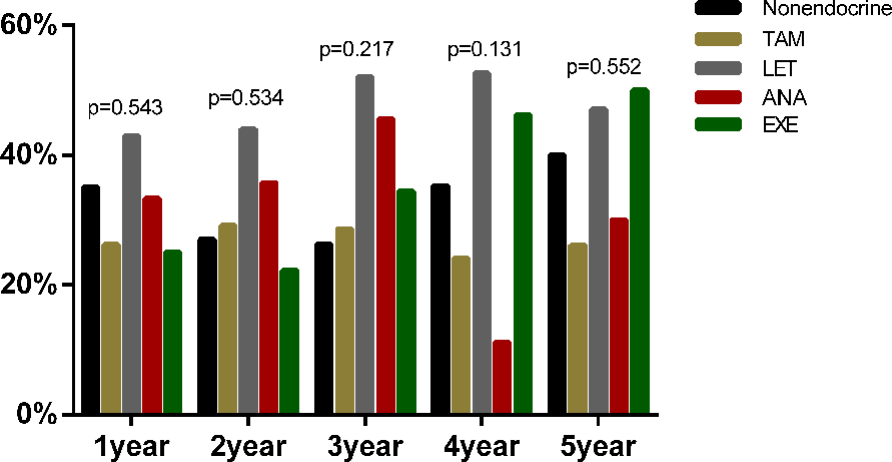
The proportion of newly diagnosed dyslipidaemia after endocrine therapy in postmenopausal BC.

Additionally, among the 731 postmenopausal patients, 521 patients were diagnosed with dyslipidaemia (71.23%) before endocrine therapy. Furthermore, we compared the proportion of newly diagnosed dyslipidaemia in patients received different endocrine drugs (**Figure 5**). Similar to the results of premenopausal patients, the proportion of newly diagnosed dyslipidaemia were also no statistical difference among nonendocrine, TAM, LET, ANA and EXE groups at all assessment time points during the whole endocrine therapy period.

### Comparison of Variations in Lipid Profiles Among Letrozole, Anastrozole and Exemestane in Postmenopausal BC Patients

To assess the effects of LET, ANA and EXE on lipid profiles, intergroup comparisons were performed. The mean absolute values for lipid parameters and the P value of pairwise comparisons across the three endocrine drugs during the study period are shown in **Table 5**. Overall, there was almost no difference in the effects of LET, ANA and EXE on lipid profiles. We found that TC levels were lower in the EXE than in the LET or ANA groups at 1 year (p=0.04; p=0.047, respectively). The LDL levels increased in the EXE group but decreased in the LET group and ANA group at 4 years (p=0.01; p=0.01, respectively).

**Table 5:** Comparision of lipid profiles among LET, ANA and EXE in postmenopausal BC patients.

	LET	N	ANA	N	EXE	N	LET vs. ANA	LET vs. EXE	ANA vs. EXE
	Lsmean (SE)		Lsmean (SE)		Lsmean (SE)		t (*P*)	t (*P*)	t (*P*)
TC									
baseline	5.11 (0.10)	151	5.16 (0.10)	118	5.20 (0.11)	82	-0.5 (0.62)	-0.71 (0.48)	-0.28 (0.78)
1 year	5.16 (0.10)	122	5.16 (0.11)	100	4.85 (0.12)	58	0 (0.99)	2.04 (0.04)	1.98 (0.047)
2 years	5.12 (0.10)	103	5.18 (0.11)	92	4.90 (0.13)	51	-0.46 (0.64)	1.46 (0.15)	1.8 (0.07)
3 years	5.09 (0.10)	94	5.18 (0.11)	82	5.02 (0.13)	43	-0.72 (0.47)	0.46 (0.64)	1.01 (0.31)
4 years	5.15 (0.11)	80	5.16 (0.11)	75	5.20 (0.14)	34	-0.1 (0.92)	-0.28 (0.78)	-0.19 (0.85)
5 years	5.21 (0.11)	62	5.17 (0.12)	59	5.06 (0.15)	24	0.32 (0.75)	0.88 (0.38)	0.62 (0.54)
TG									
baseline	1.71 (0.10)	151	1.74 (0.11)	118	1.71 (0.12)	82	-0.21 (0.84)	0.01 (0.99)	0.18 (0.86)
1 year	1.69 (0.11)	122	1.46 (0.12)	100	1.38 (0.13)	58	1.74 (0.08)	1.91 (0.06)	0.49 (0.63)
2 years	1.57 (0.11)	103	1.63 (0.12)	92	1.38 (0.14)	51	-0.48 (0.63)	1.09 (0.28)	1.46 (0.14)
3 years	1.60 (0.11)	94	1.63 (0.12)	82	1.46 (0.14)	43	-0.24 (0.81)	0.79 (0.43)	0.96 (0.34)
4 years	1.71 (0.12)	80	1.50 (0.12)	75	1.41 (0.15)	34	1.49 (0.14)	1.62 (0.10)	0.46 (0.65)
5 years	1.77 (0.12)	62	1.53 (0.13)	59	1.59 (0.16)	24	1.52 (0.13)	0.89 (0.37)	-0.31 (0.76)
HDL									
baseline	1.44 (0.17)	151	1.47 (0.19)	118	1.52 (0.22)	82	-0.15 (0.88)	-0.28 (0.78)	-0.14 (0.89)
1 year	1.60 (0.19)	122	1.68 (0.21)	100	1.50 (0.26)	58	-0.3 (0.77)	0.32 (0.75)	0.55 (0.58)
2 years	1.63 (0.21)	103	1.64 (0.21)	92	1.55 (0.27)	51	-0.03 (0.97)	0.24 (0.81)	0.26 (0.79)
3 years	1.62 (0.21)	94	1.63 (0.22)	82	1.54 (0.30)	43	-0.04 (0.97)	0.23 (0.82)	0.26 (0.80)
4 years	1.59 (0.22)	80	1.65 (0.23)	75	1.51 (0.33)	34	-0.23 (0.82)	0.19 (0.85)	0.36 (0.72)
5 years	1.59 (0.25)	62	1.64 (0.26)	59	1.43 (0.36)	24	-0.13 (0.90)	0.39 (0.70)	0.49 (0.63)
LDLchange									
1 year	-0.10 (0.09)	122	-0.21 (0.09)	100	-0.18 (0.11)	58	1.06 (0.29)	0.66 (0.51)	-0.17 (0.86)
2 years	-0.10 (0.09)	103	-0.23 (0.09)	92	-0.11 (0.11)	51	1.21 (0.23)	0.09 (0.93)	-0.85 (0.40)
3 years	-0.16 (0.09)	94	-0.23 (0.09)	82	-0.05 (0.11)	43	0.59 (0.55)	-0.81 (0.42)	-1.25 (0.21)
4 years	-0.23 (0.09)	80	-0.27 (0.10)	75	0.12 (0.12)	34	0.31 (0.76)	-2.44 (0.01)	-2.64 (0.01)
5 years	-0.17 (0.09)	62	-0.21 (0.10)	59	-0.12 (0.12)	24	0.32 (0.75)	-0.34 (0.73)	-0.59 (0.56)

## Discussion

This was a real-world retrospective study with a relatively large sample size in a single institution in China to investigate the potential effects of endocrine therapy on serum lipids. In this study, we found that for premenopausal BC patients, TAM had beneficial effects on LDL and TC levels. For postmenopausal BC patients, the effects of AIs (including LET, ANA and EXE) on lipid metabolism might be insignificant, while TAM has a modest beneficial impact on LDL.

The majority of previous studies which showed beneficial effects of TAM on LDL and TC levels were usually prospective randomized controlled trials, although the variables affecting serum lipids were relatively strictly controlled, the sample size was usually small, and the follow-up time was generally short, mostly from 3 months to 2 years (24–26). This real-world retrospective study complemented and further confirmed previous results with a relatively large sample size and longer follow-up. The beneficial effects of TAM on lower LDL levels may be explained by the fact that TAM may inhibit enzymes, including sterol-Δ8,7-isomerase and acetyl-coenzyme A acetyltransferase, which are involved in the cholesterol metabolism pathway to decrease LDL levels (27). Additionally, other research have reported that the TAM-induced decrease in LDL levels might be associated with upregulating apo B-100 receptors (28).

Regarding the effects of TAM on HDL levels, previous studies have shown different results. Wasan KM et al. reported that after 2 years of TAM administration, a moderate increase in HDL levels was observed in postmenopausal BC women (29). On the other hand, Vehmanen et al. reported that the changes in HDL cholesterol levels after 3 and 6 months of tamoxifen therapy were insignificant both in the TAM group and in the nonendocrine group (30). Similarly, another report published by Sawada also showed that TAM did not change HDL levels (24). Similarly, the present study showed that except for the lower HDL levels in the TAM group at 5 years, there was no significant difference between the TAM and nonendocrine groups.

In terms of the TG levels, different studies have different results. Tominaga et al. demonstrated that TAM did not affect TG levels (31), whereas Sawada reported that TAM increased TG by 21.7% (24). Our results showed that TAM seemed more adverse than the nonendocrine at 2 years, but the adverse effects only lasted for a short time, and there was no significant difference at other assessment time points between the TAM and nonendocrine groups. Due to the lack of long-term prospective randomized controlled trials to confirm the effects of TAM on lipid profiles, further research is urgently needed.

Regarding the effects of LET on lipid metabolism, previous studies have shown different results. Elisaf et al. have reported an increase in TC and LDL levels after the administration of LET (32). Similarlly, in the sub-study of the MA.17 trial, significant increases in TC, LDL and HDL levels were observed in the LET group compared with baseline levels, although the placebo arm was not available (33). Moreover,the first results of the BIG 1-98 trial comparing LET with TAM showed that 43.6% of LET-treated patients developed mild to moderate hypercholesterolaemia vs 19.2% of TAM-treated patients (34). While, Harper-Wynne et al. (35) and Heshmati et al. (36) demonstrated that LET had no significant effects on TC, LDL or HDL levels following 3 and 6 months of therapy. Similarly, a multicentre open randomized study containing 27 patients in the LET and TOR groups, respectively reported that after 2 years of follow-up, lipid profiles were essentially unchanged in the LET group (37). Additionally, a sub-study of the MA.17 trial (183 in the LET group and 164 in the placebo group with a 3-year follow-up) demonstrated that after 5 years of TAM treatment, LET therapy did not significantly alter TG, HDL, LDL, or TC levels, and the frequency of hypercholesterolemia was comparable between patients treated with LET and those treated with placebo (38). Overall, the current information is conflicting and insufficient to fully determine the longer-term effects of LET on lipid metabolism.

In regard to the changes in lipid profiles during the ANA treatment period, there has been much controversy. In the Italian TAM Arimidex (ITA) trial, patients switching to ANA after 2 or more years of TAM were found to have higher levels of lipid metabolism disorders than those continuing on TAM (9.3% vs 4%, respectively) (39). In addition, another study containing 38 postmenopausal BC patients showed significant increases in TC, LDL, and HDL levels (40). However, there have also been studies that showed beneficial effects on lipid profiles during the ANA treatment period. For instance, Banerjee et al. reported a significant increase in HDL levels for 176 BC patients who received 3 months of ANA treatment (41). Additionally, after 3 months of ANA treatment, a significant decrease in TG and an increase in HDL levels were observed by Sawada S (24). While, there were also studies which showed that ANA did not alter lipid profiles. Anan et al. reported a 2-year multicentre open randomized study containing 36 patients in the TOR group and 33 patients in the ANA group, and the results showed that ANA did not significantly alter lipid profiles (19). Considering the controversial results, further studies are needed to confirm the effects of ANA on lipid metabolism.

In terms of the effects of EXE on lipid metabolism, different studies have shown different results. A study showed that after 2 years of follow-up, TC, LDL, and TG levels decreased in both the EXE and placebo groups, and the only difference was a small decrease in HDL observed in the EXE group only (42). A companion sub-study of the EORTC trial (72 patients included in the statistical analysis) indicated that EXE decreased TG levels and had no adverse effects on TC or HDL levels 8, 24 and 48 weeks after treatment (43). In the Greek sub-study of the TEAM trial, baseline lipid levels were compared with levels at 3 and 6 months of treatment. At 6 months, EXE appeared to stabilize TC and HDL levels. In terms of TG levels, EXE reduced TG at both time points and by approximately 10% at 6 months (44). In brief, these studies showed that EXE seemed to have little adverse effects on lipid profiles.

Overall, few studies have explored the effects of endocrine drugs on lipid metabolism, and the results are largely controversial. Additionally, there has been no study that directly compares the effects of TAM, LET, ANA and EXE on lipid profiles in postmenopausal HR-positive BC patients. More importantly, no consensus has been reached so far. To complement previous results, we conducted a retrospective real-world analysis, and our findings indicated that there was no significant difference in the lipid profiles (TG, TC, LDL and HDL) in the LET, ANA and EXE groups compared with the nonendocrine group. For patients who received TAM, we found that LDL levels decreased from the first year, and with the increase in medication time, the effects of TAM on reducing LDL levels were weakened. In conclusion, endocrine therapy had almost no adverse effects on serum lipids in postmenopausal HR-positive BC patients. Due to the lack of long-term prospective randomized controlled trials to confirm the effects of endocrine drugs on lipid profiles, further research is urgently needed.

To the best of our knowledge, the present study was one of the first real-world retrospective studies with a 5-year follow-up and a relatively large sample size to provide comprehensive information on lipid alterations and to explore the effects of different endocrine agents on lipid profiles in both premenopausal and postmenopausal BC patients.There are still several limitations to this study that should be highlighted. First, as a retrospective study, some data were lost, and some confounding factors could not be completely excluded. Second, the present study lacks data on calorie intake and consumption, energy variation and body composition, such as dietary intake, physical activity and basic metabolic rates. Third, although the portion of patients who take hyperlipidemia drugs was less than 1%, the data was collected only from West China Hospital. It is not clear whether patients were prescribed lipid-lowering drugs in other hospitals or took drugs themselves. However, based on clinical experience, few patients took lipid-lowering drugs and thus, the results of this study were mildly affected. Additionally, many factors may have intertwining effects on lipid profiles; thus, the specific mechanism of lipid profile alterations throughout endocrine therapy was not explained clearly. Last but not least, we lacked the prognostic value of follow-up in later stages, and we did not further explore the relationship between endocrine therapy and the incidence of CVD and the long-term prognosis of BC patients. Thus, further multicenter RCT studies are urgently warranted to supplement and confirm lipid metabolism during endocrine treatment period, and the sustained effects after the end of endocrine treatment deserve increasing attention.

## Conclusion

In conclusion, favourable changes in lipid profiles, especially LDL and TC levels, were observed in premenopausal BC patients treated with TAM. Additionally, for postmenopausal BC patients, aromatase inhibitors (AIs) may have no adverse effects on lipid profiles, and TAM may have limited beneficial effects on serum lipids.

## Data Availability Statement

The original contributions presented in the study are included in the article/supplementary material. Further inquiries can be directed to the corresponding author.

## Ethics Statement

The studies involving human participants were reviewed and approved by The Institutional Review Board and Ethics Committee of West China Hospital. The patients/participants provided their written informed consent to participate in this study. Written informed consent was obtained from the individual(s) for the publication of any potentially identifiable images or data included in this article.

## Author Contributions

All authors contributed to the conception, design and interpretation of the data; the preparation of the manuscript; and the final editing and approval of the manuscript. All authors read and approved the final manuscript.

## Funding

This study was supported by Sichuan Science and Technology Program (2019YFS0338) and National Natural Science Foundation of China (32071284).

## Conflict of Interest

The authors declare that the research was conducted in the absence of any commercial or financial relationships that could be construed as a potential conflict of interest.

## Publisher’s Note

All claims expressed in this article are solely those of the authors and do not necessarily represent those of their affiliated organizations, or those of the publisher, the editors and the reviewers. Any product that may be evaluated in this article, or claim that may be made by its manufacturer, is not guaranteed or endorsed by the publisher.

## Abbreviations

BC, breast cancer; TG, triglycerides; TC, total cholesterols; LDL-C, low-density lipoprotein; HDL-C, high-density lipoprotein; CVD, cardiovascular diseases; ECOG, Eastern Cooperative Oncology Group; BMI, body mass index; DV, difference value; SERMs, selective estrogen receptor modulators; TAM, tamoxifen; AI, aromatase inhibitor; LET, letrozole; ANA, anastrozole; EXE, exemestane; TOR, toremifene.

## References

[1] Bray F Ferlay J Soerjomataram I Siegel RL Torre LA Jemal A. Global Cancer Statistics 2018: GLOBOCAN Estimates of Incidence and Mortality Worldwide for 36 Cancers in 185 Countries. CA Cancer J Clin (2018) 68(6):394–424. doi: 10.3322/caac.21492 30207593

[2] Li H Zheng RS Zhang SW Zeng HM Sun KX Xia CF. Incidence and Mortality of Female Breast Cancer in China, 2014. Zhonghua Zhong Liu Za Zhi (2018) 40(3):166–71. doi: 10.3760/cma.j.issn.0253-3766.2018.03.002 29575833

[3] Bernstein L Lacey JV Jr. Receptors, Associations, and Risk Factor Differences by Breast Cancer Subtypes: Positive or Negative? J Natl Cancer Inst (2011) 103(6):451–3. doi: 10.1093/jnci/djr046 21346225

[4] Chen WY Colditz GA. Risk Factors and Hormone-Receptor Status: Epidemiology, Risk-Prediction Models and Treatment Implications for Breast Cancer. Nat Clin Pract Oncol (2007) 4(7):415–23. doi: 10.1038/ncponc0851 17597706

[5] Perou CM Sørlie T Eisen MB van de Rijn M Jeffrey SS Rees CA. Molecular Portraits of Human Breast Tumours. Nature (2000) 406(6797):747–52. doi: 10.1038/35021093 10963602

[6] Dubsky P Filipits M Jakesz R Rudas M Singer CF Greil R. EndoPredict Improves the Prognostic Classification Derived From Common Clinical Guidelines in ER-Positive, HER2-Negative Early Breast Cancer. Ann Oncol (2013) 24(3):640–7. doi: 10.1093/annonc/mds334 PMC3574544 23035151

[7] Klinge CM. Estrogen Receptor Interaction With Estrogen Response Elements. Nucleic Acids Res (2001) 29(14):2905–19. doi: 10.1093/nar/29.14.2905 PMC55815 11452016

[8] Platet N Cathiard AM Gleizes M Garcia M. Estrogens and Their Receptors in Breast Cancer Progression: A Dual Role in Cancer Proliferation and Invasion. Crit Rev Oncol Hematol (2004) 51(1):55–67. doi: 10.1016/j.critrevonc.2004.02.001 15207254

[9] Pickar JH MacNeil T Ohleth K. SERMs: Progress and Future Perspectives. Maturitas (2010) 67(2):129–38. doi: 10.1016/j.maturitas.2010.05.009 20580502

[10] Yu KD Zhou Y Liu GY Li B He PQ Zhang HW. A Prospective, Multicenter, Controlled, Observational Study to Evaluate the Efficacy of a Patient Support Program in Improving Patients’ Persistence to Adjuvant Aromatase Inhibitor Medication for Postmenopausal, Early Stage Breast Cancer. Breast Cancer Res Treat (2012) 134(1):307–13. doi: 10.1007/s10549-012-2059-8 22527106

[11] Grilli S. Tamoxifen (TAM): The Dispute Goes on. Ann Ist Super Sanita (2006) 42(2):170–3. 17033137

[12] Murphy MJ Jr. Molecular Action and Clinical Relevance of Aromatase Inhibitors. Oncologist (1998) 3(2):129–30. doi: 10.1634/theoncologist.3-2-129 10388095

[13] Dowsett M. Aromatase Inhibitors Come of Age. Ann Oncol (1997) 8(7):631–2. doi: 10.1023/a:1008282315089 9296213

[14] Goss PE. Risks Versus Benefits in the Clinical Application of Aromatase Inhibitors. Endocr Relat Cancer (1999) 6(2):325–32. doi: 10.1677/erc.0.0060325 10731126

[15] Davies C Pan H Godwin J Gray R Arriagada R Raina V. Long-Term Effects of Continuing Adjuvant Tamoxifen to 10 Years Versus Stopping at 5 Years After Diagnosis of Oestrogen Receptor-Positive Breast Cancer: ATLAS, A Randomised Trial. Lancet (2013) 381(9869):805–16. doi: 10.1016/S0140-6736(12)61963-1 PMC3596060 23219286

[16] Iorga A Cunningham CM Moazeni S Ruffenach G Umar S Eghbali M. The Protective Role of Estrogen and Estrogen Receptors in Cardiovascular Disease and the Controversial Use of Estrogen Therapy. Biol Sex Differ (2017) 8(1):33. doi: 10.1186/s13293-017-0152-8 29065927 PMC5655818

[17] Schenck-Gustafsson K. Risk Factors for Cardiovascular Disease in Women: Assessment and Management. Eur Heart J (1996) 17 Suppl D:2–8. doi: 10.1093/eurheartj/17.suppl_d.2 8869875

[18] Patnaik JL Byers T DiGuiseppi C Dabelea D Denberg TD. Cardiovascular Disease Competes With Breast Cancer as the Leading Cause of Death for Older Females Diagnosed With Breast Cancer: A Retrospective Cohort Study. Breast Cancer Res (2011) 13(3):R64. doi: 10.1186/bcr2901 21689398 PMC3218953

[19] Anan K Mitsuyama S Yanagita Y Kimura M Doihara H Komaki K. Effects of Toremifene and Anastrozole on Serum Lipids and Bone Metabolism in Postmenopausal Females With Estrogen Receptor-Positive Breast Cancer: The Results of a 2-Year Multicenter Open Randomized Study. Breast Cancer Res Treat (2011) 128(3):775–81. doi: 10.1007/s10549-011-1608-x 21638048

[20] Hozumi Y Suemasu K Takei H Aihara T Takehara M Saito T. The Effect of Exemestane, Anastrozole, and Tamoxifen on Lipid Profiles in Japanese Postmenopausal Early Breast Cancer Patients: Final Results of National Surgical Adjuvant Study BC 04, the TEAM Japan Sub-Study. Ann Oncol (2011) 22(8):1777–82. doi: 10.1093/annonc/mdq707 21285133

[21] Bell LN Nguyen AT Li L Desta Z Henry NL Hayes DF. Comparison of Changes in the Lipid Profile of Postmenopausal Women With Early Stage Breast Cancer Treated With Exemestane or Letrozole. J Clin Pharmacol (2012) 52(12):1852–60. doi: 10.1177/0091270011424153 PMC3616612 22174434

[22] Gradishar WJ Anderson BO Balassanian R Blair SL Burstein HJ Cyr A. Breast Cancer, Version 4.2017, NCCN Clinical Practice Guidelines in Oncology. J Natl Compr Canc Netw (2018) 16(3):310–20. doi: 10.6004/jnccn.2018.0012 29523670

[23] Joint committee issued Chinese guideline for the management of dyslipidemia in adults. 2016 Chinese Guideline for the Management of Dyslipidemia in Adults. Zhonghua Xin Xue Guan Bing Za Zhi (2016) 44(10):833–53. doi: 10.3760/cma.j.issn.0253-3758.2016.10.005 27903370

[24] Sawada S Sato K Kusuhara M Ayaori M Yonemura A Tamaki K. Effect of Anastrozole and Tamoxifen on Lipid Metabolism in Japanese Postmenopausal Women With Early Breast Cancer. Acta Oncol (2005) 44(2):134–41. doi: 10.1080/02841860510007585 15788292

[25] Decensi A Robertson C Rotmensz N Severi G Maisonneuve P Sacchini V. Effect of Tamoxifen and Transdermal Hormone Replacement Therapy on Cardiovascular Risk Factors in a Prevention Trial. Ital Chemoprevention Group Br J Cancer (1998) 78(5):572–8. doi: 10.1038/bjc.1998.542 PMC2063049 9744493

[26] Markopoulos C Polychronis A Dafni U Koukouras D Zobolas V Tzorakoleftherakis E. Lipid Changes in Breast Cancer Patients on Exemestane Treatment: Final Results of the TEAM Greek Substudy. Ann Oncol (2009) 20(1):49–55. doi: 10.1093/annonc/mdn545 18678766

[27] Grainger DJ Schofield PM. Tamoxifen for the Prevention of Myocardial Infarction in Humans: Preclinical and Early Clinical Evidence. Circulation (2005) 112(19):3018–24. doi: 10.1161/CIRCULATIONAHA.104.531178 16275887

[28] Stevenson JC. Mechanisms Whereby Oestrogens Influence Arterial Health. Eur J Obstet Gynecol Reprod Biol (1996) 65(1):39–42. doi: 10.1016/0028-2243(95)02301-8 8706955

[29] Wasan KM Ramaswamy M Haley J Dunn BP. Administration of Long-Term Tamoxifen Therapy Modifies the Plasma Lipoprotein-Lipid Concentration and Lipid Transfer Protein I Activity in Postmenopausal Women With Breast Cancer. J Pharm Sci (1997) 86(7):876–9. doi: 10.1021/js970097w 9232532

[30] Vehmanen L Saarto T Blomqvist C Taskinen MR Elomaa I. Tamoxifen Treatment Reverses the Adverse Effects of Chemotherapy-Induced Ovarian Failure on Serum Lipids. Br J Cancer (2004) 91(3):476–81. doi: 10.1038/sj.bjc.6601979 PMC2409844 15266329

[31] Tominaga T Kimijima I Kimura M Takatsuka Y Takashima S Nomura Y. Effects of Toremifene and Tamoxifen on Lipid Profiles in Post-Menopausal Patients With Early Breast Cancer: Interim Results From a Japanese Phase III Trial. Jpn J Clin Oncol (2010) 40(7):627–33. doi: 10.1093/jjco/hyq021 20382637

[32] Elisaf M Bairaktari E Nicolaides C Fountzilas G Tzallas C Siamopoulos K. The Beneficial Effect of Tamoxifen on Serum Lipoprotein-A Levels: An Additional Anti-Atherogenic Property. Anticancer Res (1996) 16(5A):2725–8. 8917378

[33] Wasan KM Goss PE Pritchard PH Shepherd L Tu D Ingle JN. Lipid Concentrations in Postmenopausal Women on Letrozole After 5 Years of Tamoxifen: An NCIC CTG MA.17 Sub-Study. Breast Cancer Res Treat (2012) 136(3):769–76. doi: 10.1007/s10549-012-2294-z 23089983

[34] Breast International Group (BIG) 1-98 Collaborative Group Thürlimann B Keshaviah A Coates AS Mouridsen H Mauriac L. A Comparison of Letrozole and Tamoxifen in Postmenopausal Women With Early Breast Cancer. N Engl J Med (2005) 353(26):2747–57. doi: 10.1056/NEJMoa052258 16382061

[35] Harper-Wynne C Ross G Sacks N Salter J Nasiri N Iqbal J. Effects of the Aromatase Inhibitor Letrozole on Normal Breast Epithelial Cell Proliferation and Metabolic Indices in Postmenopausal Women: A Pilot Study for Breast Cancer Prevention. Cancer Epidemiol Biomarkers Prev (2002) 11(7):614–21. 12101108

[36] Heshmati HM Khosla S Robins SP O’Fallon WM Melton LJ 3rd Riggs BL. Role of Low Levels of Endogenous Estrogen in Regulation of Bone Resorption in Late Postmenopausal Women. J Bone Miner Res (2002) 17(1):172–8. doi: 10.1359/jbmr.2002.17.1.172 11771665

[37] Shien T Doihara H Sato N Anan K Komaki K Miyauchi K. Serum Lipid and Bone Metabolism Effects of Toremifene vs. Letrozole as Adjuvant Therapy for Postmenopausal Early Breast Cancer Patients: Results of a Multicenter Open Randomized Study. Cancer Chemother Pharmacol (2018) 81(2):269–75. doi: 10.1007/s00280-017-3491-6 PMC5778152 29196963

[38] Wasan KM Goss PE Pritchard PH Shepherd L Palmer MJ Liu S. The Influence of Letrozole on Serum Lipid Concentrations in Postmenopausal Women With Primary Breast Cancer Who Have Completed 5 Years of Adjuvant Tamoxifen (NCIC CTG MA.17l). Ann Oncol (2005) 16(5):707–15. doi: 10.1093/annonc/mdi158 15817595

[39] Boccardo F Rubagotti A Puntoni M Guglielmini P Amoroso D Fini A. Switching to Anastrozole Versus Continued Tamoxifen Treatment of Early Breast Cancer: Preliminary Results of the Italian Tamoxifen Anastrozole Trial. J Clin Oncol (2005) 23(22):5138–47. doi: 10.1200/JCO.2005.04.120 16009955

[40] Hozumi Y Saito T Inoue K Shiozawa M Omoto Y Tabei T. Effects of Anastrozole on the Lipid Profile in Postmenopausal Breast Cancer Patients - A Preliminary Study. In: Fourth European Breast Cancer Conference. Hamburg, Germany, Abstract 300. (2004)

[41] Banerjee S Smith IE Folkerd L Iqbal J Barker P Dowsett M. Comparative Effects of Anastrozole, Tamoxifen Alone and in Combination on Plasma Lipids and Bone-Derived Resorption During Neoadjuvant Therapy in the Impact Trial. Ann Oncol (2005) 16(10):1632–8. doi: 10.1093/annonc/mdi322 16030027

[42] Lønning PE Geisler J Krag LE Erikstein B Bremnes Y Hagen AI. Effects of Exemestane Administered for 2 Years Versus Placebo on Bone Mineral Density, Bone Biomarkers, and Plasma Lipids in Patients With Surgically Resected Early Breast Cancer. J Clin Oncol (2005) 23(22):5126–37. doi: 10.1200/JCO.2005.07.097 15983390

[43] Atalay G Dirix L Biganzoli L Beex L Nooij M Cameron D. The Effect of Exemestane on Serum Lipid Profile in Postmenopausal Women With Metastatic Breast Cancer: A Companion Study to EORTC Trial 10951, ‘Randomized Phase II Study in First Line Hormonal Treatment for Metastatic Breast Cancer With Exemestane or Tamoxifen in Postmenopausal Patients’. Ann Oncol (2004) 15(2):211–7. doi: 10.1093/annonc/mdh064 14760111

[44] Markopoulos C Polychronis A Zobolas V Xepapadakis G Papadiamantis J Koukouras D. The Effect of Exemestane on the Lipidemic Profile of Postmenopausal Early Breast Cancer Patients: Preliminary Results of the TEAM Greek Sub-Study. Breast Cancer Res Treat (2005) 93(1):61–6. doi: 10.1007/s10549-005-3783-0 16184460

